# Supplementary material for: A retrospective review on antibiotic use in acute watery diarrhea in children in a tertiary care hospital of Karachi, Pakistan
Source: PLoS One. 2021 Jul 15;16(7):e0253712. doi: 10.1371/journal.pone.0253712 (PMC8282082; doi:10.1371/journal.pone.0253712)
Supplement: Attachment — Submitted filename: Response to Reviewers.docx [file pone.0253712.s001.docx]

**Point-by-point response to the reviewers**

**Submission Ref. No:** **PONE-D-21-04700**

**Title:** Association between antibiotic use and length of hospital stay among children presented with acute watery diarrhea in a tertiary care hospital, Karachi. A Retrospective study

I am thankful to all reviewers for providing valuable input on this manuscript. In light of your suggestions manuscript has been amended and responses to mentioned points are given below:

| **Comments** | **Responses** |
| --- | --- |
| **Reviewer#1:** |  |
| 1. The authors should provide more details on the methods of medical records review. What is the system for medical recording in this hospital? Is it electronic or paper based? Is “acute watery diarrhea” an agreed diagnostic term or has a specific code to be recorded? Who reviewed the records (a single person? Two?)? Was there a double check or revision? …. etc | The system of recording at the hospital is paper-based and a digital record is also maintained for diagnosis from which data is extracted by using ICD codes.  We pulled out medical record numbers of all children admitted with acute gastroenteritis during the defined time period and those fulfilling the pre-defined clinical definition were enrolled. |
| 2. Authors should provide the indications of hospital admission for cases included in this study, what were the guidelines for antibiotics prescription in acute watery diarrhea in this hospital (39.4% of children with acute watery diarrhea who have no dysentery, no chronic illness, and no immunocompromised status received antibiotics), what were the guidelines for giving intravenous fluids (as need for intravenous rehydration after 24 hours of hospital admission was one of the study outcomes, and what were the discharge criteria (as length of hospital stay is the primary outcome). Is it possible that children who received the antibiotics stayed for a longer time in the hospital in order to complete a certain course of antibiotics? | There is no local guideline. We usually follow the standard guidelines but decision to prescribe antibiotics depends  upon the severity of underlying illness and at the physician’s discretion. Similarly IV rehydration is provided if child has severe loses or doesn’t tolerate orally. |
| 3. Authors should provide methods for assessment of the severity of dehydration, | The clinical assessment for severity of illness is based on skin turgor, eagerness to drink, sunken eyes and irritability as defined by  WHO IMCI (Integrated Management of Childhood Illness) chart.  We follow these four criteria for dehydration severity grading in our hospital. As it is a retrospective review, we collected documented information from medical record file of each patient at the time of presentation. |
| 4. The authors conclude that antibiotic use was associated with a prolonged hospital stay in children with acute watery diarrhea. They report that the expected length of hospital stay for the antibiotic group was 0.22 hours higher than the group that did not receive antibiotics. However, even if this finding is statistically significant, I am concerned about the clinical importance with this small effect size (0.22 hours). | Thank you for highlighting this point. We understand this limitation. This may be because of small sample size. |
| 5. In the discussion, authors should explain the possible reasons/mechanisms for their findings, such as the association of antibiotic use and increased length of hospital stay and the association between male gender and longer hospital of stay. | This is really an important point which we missed in our manuscript to discuss.  Our findings indicated that antibiotic use was associated with a longer hospital stay. This may be because more children (7.3%) in the antibiotic group presented with severe dehydration compared to the non-antibiotic group (1.9%). Similarly 74/116 (63.7%) of male patients had signs of dehydration on presentation compared to females 35/59, (59.3%) or because in developing countries where illiteracy rate is high , families are more prone to insist on prolonged admission of their male heirs as compared to females. This can be a major contributing factor in the difference in results, especially when viewed as a component of gender inequality.  We have added the reasoning in the modified version of the manuscript (Line no: 215-216, 225-231, 234-241) |
| 6. The authors incorrectly use “cross-sectional study” instead of “retrospective study” in the title and methods. | This query has been addressed in the updated manuscript and the title has been edited. |
| **Reviewer#2:** |  |
| 1. The Language needs to be more polished, and correction of some grammatical errors is essential | This query has been addressed by an in-depth analysis of the paper and corrections in the updated manuscript. |
