# Supplementary material for: A retrospective review on antibiotic use in acute watery diarrhea in children in a tertiary care hospital of Karachi, Pakistan
Source: PLoS One. 2021 Jul 15;16(7):e0253712. doi: 10.1371/journal.pone.0253712 (PMC8282082; doi:10.1371/journal.pone.0253712)
Supplement: Attachment — Submitted filename: Response to Reviewers.docx [file pone.0253712.s002.docx]

**Point-by-point response to the reviewers**

**Submission Ref. No:** **PONE-D-21-04700**

**Title:** Association between antibiotic use and length of hospital stay among children presented with acute watery diarrhea in a tertiary care hospital, Karachi. A Retrospective study

I am thankful to all reviewers for providing valuable input on this manuscript. In light of your suggestions manuscript has been amended and responses to mentioned points are given below:

| **Comments** | **Responses** |
| --- | --- |
| **Reviewer#3:** |  |
| 1. P6, L112: Is it a cross-section study or retrospective study? | We are very sorry that we forgot to do this change in the study manuscript  We have done the corrections in the revised manuscript (P5, L103).  Sorry for the inconvenience |
| 2. The indication for antibiotic use or admission should be provided to make the result or conclusion more convincing. Or this could be the major limitation in your study | There is no local hospital guideline. We usually follow the standard guideline. The common practice in our institute to prescribe antibiotics depends upon the severity of underlying illness and at the **physician’s discretion**.  This is the basis of conducting this study to increase the awareness of our physicians that all should stick and follow the standard guidelines and if diverting from the pathway there must be some rationale of starting the antibiotics as in acute watery diarrhea there is no role of antibiotics.  Similarly most common reasons for admission in our hospital are unable to tolerate feed, dehydration and parental anxiety.  As this is the retrospective review this information is difficult to collect.  This study helps us in developing a local hospital guideline and improving the knowledge of our HCWS so that everyone follow that standard pathway and in this way we can able to control the unnecessary use of antibiotics in our setting and in settings where such practice follows by disseminating the results of our study. |
| 3. The association between antibiotics use and prolonged hospitalization was not explained well in the discussion. In different settings, antibiotic choices, different background, all variables will change. In limited information from limited cases, the association was not strong enough to make the conclusion. Try to find more variables, such as categories of antibiotics, pathogens, might be helpful. | We have mentioned the categories of antibiotics prescribed in our manuscript. Very few stool cultures came out to be positive. The description of which is given in the write-up. We know that the yield of stool culture to detect a bug is very low particularly in the setting where self-medication and overuse of antibiotics are common practice in the community.  We agree with you that this association is not strong enough to make conclusion. To answer this question we need more large scale studies which we recommended in our conclusion. This study will help in developing the background/rationale to design large scale multicenter studies/trials. We have also mentioned/modified our recommendation in the abstract and in main text (P3, L51-52, P19, L276-277)).  We hope we have responded your query appropriately. |
| 4. I have the same question about the clinical significance of “0.22 hour” longer in hospitalization as previous reviewer stated. It has minimal drive for clinician to change their daily practice | Thank you for highlighting this point. We have elaborated this point in comment 3.  We understand this limitation. Therefore we recommend larger studies in our conclusion. |
| 5. Virus should not be italic or uppercase compared with bacteria. Ex: Diarrhea caused by “rotavirus” and “Campylobacter jejuni” | Thank you for picking this up. We have made corrections as suggested (P4, L82). |
| **Reviewer#4:** |  |
| 1. Methods: The diagnosis criteria of acute watery diarrhea were not defined. The authors described only exclusion criteria. The authors did stool culture (Table 1. Stool culture positive rate). Please describe the inclusion criteria and the antibiotics category and duration | Inclusion criteria is elaborated in methods section (P5, L106-108).  Antibiotics categories and median duration of antibiotics are mentioned in the text of result section (P6, L146-149).  Details of positive stool cultures are mentioned in the text of result section (P8, L155-159). |
| 2. Antibiotic therapy is not necessary for acute diarrhea in children because rehydration is the crucial treatment and symptoms resolve without specific therapy. Please describe the indication for antibiotics in this manuscript. | Same as response 2 mentioned in reviewer 3 comments. |
| 3. Methods, page 112: The phrase “A cross-sectional study was conducted at the department of Pediatric---” was not corrected in the revised manuscript. | Thankyou so much for highlighting this. We have corrected it in the revised manuscript (P5, L103). |
| 4. Figure 1: Please describe the number of the enrolled participants in each group. | We have added this information in the Figure. Kindly refer to the revised version. |
