# Supplementary material for: A retrospective review on antibiotic use in acute watery diarrhea in children in a tertiary care hospital of Karachi, Pakistan
Source: PLoS One. 2021 Jul 15;16(7):e0253712. doi: 10.1371/journal.pone.0253712 (PMC8282082; doi:10.1371/journal.pone.0253712)
Supplement: Attachment — Submitted filename: Response to Reviewers.docx [file pone.0253712.s003.docx]

**Point-by-point response to the reviewers**

**Submission Ref. No:** **PONE-D-21-04700**

**Title:** A retrospective review on antibiotic use in acute watery diarrhea in children in a tertiary care hospital, Karachi

I am thankful to all reviewers for providing valuable input on this manuscript. In light of your suggestions manuscript has been amended and responses to mentioned points are given below:

| **Comments** | **Responses** |
| --- | --- |
| 1. Please revise the Title to make it concise whereas informative | We have revised the title as suggested. Kindly refer to the revised manuscript. |
