# Supplementary material for: A retrospective review on antibiotic use in acute watery diarrhea in children in a tertiary care hospital of Karachi, Pakistan
Source: PLoS One. 2021 Jul 15;16(7):e0253712. doi: 10.1371/journal.pone.0253712 (PMC8282082; doi:10.1371/journal.pone.0253712)
Supplement: Attachment — Submitted filename: Response to Reviewers.docx [file pone.0253712.s005.docx]

**Point-by-point response to the reviewers**

**Submission Ref. No:** **PONE-D-21-04700**

**Title:** A retrospective review on antibiotic use in acute watery diarrhea in children in a tertiary care hospital of Karachi, Pakistan.

I am thankful to all reviewers for providing valuable input on this manuscript. In light of your suggestions manuscript has been amended and responses to mentioned points are given below:

| **Comments** | **Responses** |
| --- | --- |
| **Reviewer 5** | |
| 1. It is professionally written paper on one of important public health issue of developing countries. I suggest excluding table 3 and table 4 as both intravenous fluid and diarrheal episodes are not primary objectives of the report. Table 2 is sufficient to present association of clinical manifestations with hospital stay | Thank you for your suggestion. We agree that table 3 and 4 are covering our secondary objectives. We request you to kindly keep it in the manuscript as they were our secondary objectives (Refer to highlighted part on page 5 of revised manuscript). These tables provide additional findings related to antibiotic use in acute watery diarrhea and number of diarrheal episodes and need of IV rehydration. They are adding further strength to our paper.  If still reviewer wants to remove these tables we will remove them. |
